# The Hospital Nursing Supplement

**Published:** 1893-05-13

**Authors:** 


					TllS Hospital, May 13, 1893. Extra Supplement.
" Huts tug Attvvor
Being ?hb Extba Nubsinq Supplement oj "The Hospital" Newspapeb,
[Contributions for thin Supplement should be addressed to the Editor, The Hospital, 428, Strand, London, W.O., and should hare tha word
" Nursing" plainly written in left-hand top corner of the envelope.]
1Flews from tbe IRurstna MorlD.
MINERS AT WINDSOR.
A most interesting scene took place in Windsor Park
on Saturday last, when the Queen inspected a colliery
ambulance corps composed of miners from the counties
of Derby and Nottingham Colonel Seeley employs
4.000 men in his mines, and 1,323 of these hold certifi-
cates from the St. John's Ambulance Association,
whilst 110 of the wives and daughters possess first-aid
certificates. About 350 of these men were present at
Windsor, and an excellent display took place, the Queen
showing great interest in the skill with which the
miners lifted and removed certain members of the com-
pany whom they had previously bandaged and tended
for imaginary injuries with amazing gentleness. The
hospital tent, and its band of ambulance nurses, were
afterwards inspected, and the Queen addressed a few
sympathetic words to the women, and Colonel and Mrs.
Seeley and Mrs. Wardle were afterwards presented to
her Majesty.
DEFINED DUTIES.
A memorial has been forwarded to the Local
Government Board by the Workhouse Infirmary
Nursing Association, dealing with questions arising
from the recent growth of sick nursing in the institu-
tions under the control of the Board. The increased
and systematic care given to the sick poor, and the
employment of competent trained nurses in workhouse
infirmaries, appear to call for a more accurate defini-
tion of " the duties of the officers concerned, and their
responsibilities towards the sick and each other."
Doubtless the Board which has offered such marked
encouragement to a long-needed reform, will be found
willing to give its best consideration to a question on
which the successful working of the new order of
things entirely depends.
THE INVALID CHILDREN'S AID ASSOCIATION.
There are now 2,200 names on the books of the
Invalid Children's Aid Association, 18, Buckingham
Street, Strand, and cach entry represents a young
creature who still needs, or who has received, assist-
ance?sometimes a long sojourn in a convalescent
home is the requirement, or the gift of a surgical in-
strument, or the loan of a spinal carriage or wheel-
chair. Either of these costs a good deal of money ;
in fact, the I.C.A.A. is always being requested to
supply something or other towards; which a very small
contribution, and sometimes none at all, can be made
by the applicants. To help sick and ciippled children
in their own homes is assuredly a work to be encouraged,
and it is with surprise as well as regret that we read in
the report, " with an annual expenditure exceeding two
thousand pounds, we began 1893 with ?10 17s. 9d."
THE NURSE DOLLS.
The Nurse Dolls have accepted an invitation to the
exhibition which is to be held at the Mansion, Victoria
Park, Keighley, next June, and they are asked to
Blackpool in September. As the proceeds of the
former are to go to tbe Cottage Hospital, it is specially
appropriate for the doHs to give their services on the
occasion. The Blackpool and Keighley Committees
have arranged to re-dress and renovate them. How
many of the correspondents who made such kind offers
lately to help with the dolls will assist us instead to
add to The Hospital Collection of nursing uniforms ?
We want a sample of a pretty and suitable Cottage
Hospital outfit, and we shall be glad to hear from any
readers who are willing to send dolls dressed in the
following uniforms, as we wish to have the collection
made as complete as possible: Great Yarmouth Hos-
pital, Guildford (Royal Surrey1 County), Halifax In-
firmary, Hartlepool Hospital, Hastings (East Sussex
Hospital), Hemel Hempstead Infirmary, Hereford and
Hull Infirmaries, Huntingdon and Ipswich Hospitals,
Jersey Infirmary, Leamington Hospital, Leeds General
Infirmary, Maidstone Hospital, Manchester Southern,
Margate Sea-Bathing Infirmary, Mertbyr General
Hospital, Middlesbrough and Northampton Infirmaries,
Newcastle Royal Infirmary, and the Fleming Memorial
Children's Hospital.
RULES.
The schoolmaster's query, "What do you think rules
are made for ? " and the pupil's concise reply, " Please,
sir, to be broken ! " is a saying familiar to most of us.
But, however strong her objections to foolish regula-
tions, no nurse would ever expect an institution to be
satisfactorily managed without a proper code of rules
for the guidance of the Btaff. Every well-managed
household follows a certain routine, and in all business
establishments definite orders are given ; and yet a mem-
ber of a north-couniry House Committee has recently
taken exception to there being "rules" for hospital
nurses. Not being a matron nor even a trained nurse,
he fails to see why probationers cannot absent them-
selves from the institution without leave, nor why it is
undesirable for them either to alter their hours of
recreation or to go into wards where they are not
working without permission from the matron. Before
taking exception to such simple and necessary regula-
tions, it would be well to inquire into their origin, and
at least to credit the heads of the nursing schools with
some elementary knowledge of the subject to which
they have devoted so many years?viz., the best methods
of training and governing probationers.
DANGEROUS PLAYGROUNDS.
Many a mother and many a nurse view with serenity
the small children in their charge when they play upon
the floor. Even an infant just beginning to crawl is
considered "quite safe" when he is deposited there,either
with or without a blanket. Certainly the little ones are,
for the moment, protected from the possibilities of
falls from chairs or other articles of furniture, but they
are exposed to many invisible dangers. Floors are
generally cold, even when the carpet is thick and warm.
There must be always draughts under the doors, which
lxii THE HOSPITAL NURSING SUPPLEMENT\ May 13, 1893
are specially disastrous in their effects on the tender
little bodies, seldom hardy enough to bear such trials
?with impunity. A thick rug on a grass plot is a safer
playground oftimes than the floor of a nursery where
the woodwork is defective and the sun does not pene-
trate. The charming public parks at Paris have lately
been accused of fostering and spreading deadly
fevers amongst the dainty juveniles who frequent
them, various causes being assigned for this.
Altogether the dangers of play places apparently
well suited to the little ones who frequent them, are
criticised freely. Watchfulness and incessant care on
the part of the guardians of children are, after all, the
only precautionary measures to be recommended. Each
woman's intelligent observation can do more in the
matter than any hard and fast rules laid down for he-.
VOLUNTARY HELP.
Most institutions have some special characteristic,
either some detail of management, or a particular de-
partment which singles out each, from others of the
same kind. The Hospital for Incurable Children at 2,
Maida. Yale, has a rather unique plan, of voluntary
workers. It is organised in an admirably methodical
fashion by the Matron, and a list of the ladies appears
in the annual report. This shows that these regular
visitors "amuse and instruct the little patients,"
teach them needlework, &c. Other ladies attend in
turn, and work in the wards for certain hours daily. In
giving their services, they also learn a great many
useful nursing details. The Matron is a qualified and
experienced nurse, and she and iher resident assistants
give to their lady-helpers valuable instruction. There
is eo much work wasted in this world by would-be
charitable people, who do not know how best to apply
their energies and leisure, to help the over-worked and
the needy that it is refreshing to meet with a Home like
the one in Maida Yale, where outside assistance is
utilised in a systematic manner, which reflects the
highest credit on the head of the nursing department,
as well as on her excellent contingent of voluntary '
helpers themselves. Although a good list of ladies'
names adorns the report, our readers need not imagine
that fresh workers are no longer r( quired. The fact
is that constant changers occur amongst voluntary
helpers, and therefore we believe that Miss Coleman is
always glad to have the names and addresses of young
ladies willing to aid her poor little folks, to whose
afflictions the sad word " incurable," unhappily
belongs.
UNREASONABLE OBJECTIONS.
Y.ERY much to their credit, the nurses at St.
George's-in-the-East Infirmary wish to have a library.
A great many hospitals make a special feature of a
" Nurses' Library," and by subscriptions and donations
it is increased and maintained. The workers at St.
George's have only asked the Board for their " sanc-
tion "; in other words, they have meekly requested to
be allowed to keep a collection of books, purchased with
their own money. The Matron, whose interest in her
nurses is well known, ventured to suggest to the Board
that a donation from themselves "would be appre-
ciated and this reasonable proposal was very proDerly
complied with. One guardian, however, seemed to
think they might want a carriage and pair next! We
hardly think that nurses in an infirmary would waste
their breath in demanding impossibilities, although we
own that an occasional drive, free of expense, is about
the most healthful relaxation which can be offered to
weary women. But guardians should not confound
reading and self-indulgence. A desire for wisely-
chcsen books proves the existence of intelligent aspira-
tions, which should be gladly fostered by committees.
The Board at St. George's-in-the-East finally sanc-
tioned the formation of a library, and gave permis-
sion to the nurses to spend some of their well-earned
money on books for their general benefit. We shall
be glad if our readers suggest to friends that gifts of
books are always acceptable to matrons, nurses' as well
as patients' libraries standing in constant need of re-
plenishment.
A CONVALESCENT IN GAOL.
A patient who had been in West Bromwich
Infectious Diseases Hospital for four or five weeks took
it into his head the other day to discharge himself from
the institution. He scaled a wall, says the Medical
Press, and departed with a suit of clothes belonging to
another patient, and was eventually arrested and sent
to gaol for seven days with hard labour. Where were
the nurses, and by whose authority are the clothes of a
recently-admitted small-pox case left in reach of other
patients ? Such an ai-rangement would not be sanc-
tioned in a general hospital, and in an institution for
the treatment of infectious diseases it certainly calls for
some explanation. It is also strange that a man
capable of hard labour should be detained in a hospital,
as he seems have been considered non-infectious, other-
wise the magistrates would hardly have risked the
transfer to gaol.
PARISIAN PRECOCITY.
The movement in Paris to allow pupils oUycees who
have attained the age of eighteen to inscribe them-
selves on an authorised list as " voluntary nurses," can
hardly meet with universal approbation. Courses of
instruction for these young people are being organised
by Dr. Duchauasey, and the Minister of War and the
Minister of Public Instruction have sanctioned the
movement. The earlier young people learn to give
" first aid" to any sick folks, the better for them-
selves and their neighbours, but we can hardly think
these immature lycee pupils should be authorised to
dress wounds and attend to the sick and wounded in
time of war. It is a little hard on the soldiers who risk
their lives for their country that they should have to
face a new danger which appears in the guise of
volunteer amateur nurses. Surely the word " nurses "
is misused altogether in this connection.
LEPERS IN NEW SOUTH WALES.
At the Little Bay Lazaret, New South Wales, the
patients suffering from leprosy are being " well
attended to," says the Aiistralian Star, "by an experi-
enced wardsman in charge, two male attendants and
two female nurses, under the direct supervision of the
medical superintendent and matron of the Coast
Hospital."
THE DISABLED NURSE.
The Disabled Nurse writes : I am glad to say I feel
a little better, although I do not seem to gain strength.
We have to acknowledge 5s. from F. S. ; making
?14 Os. 6d. received up to date, of which ?0 17a. Cd.
has already been handed to the invalid.
"THE HOSPITAL" ENDOWED BED.
We beg to remind our readers that many subscrip-
tions to The Hospital Endowed Bed are still due. We
have received ?1 Is. from H. L. WM with a kind letter.
Total amount up to present date, ?18 19s. Cd.
May 13, 1893. THE H0SP11AL NURSING SUPPLEMENT. lxiii
Sanitation for Burses.
By a Sanitary Inspector.
V.~NOTIFICATION IN FOREIGN COUNTRIES.
ITALY.
In Italy we find the chief control of public health rests
with the Minister of the Interior, and under him are the
Prefects, Sub-Prefects, and Syndics, which last are the
equivalent of the French "Maires." Thus whilst there is
one Central Council of Health in the capital, each province
is provided with a Provincial Council and a Medical Officer
of Health. Then in every Commune, or sub-division of the
province, there is a medical officer to superintend local sani-
tary matters. Whenever a case of infectious disease
appears, it is required that the doctor in attendance should
immediately notify it to both the Syndic and also to the
Medical Officer of Health of the Commune ; and the Syndic
notifies to the Prefect and he to the Minister of the Interior.
The doctor in attendance co-operates with the Syndic and
Communal sanitary authority, carrying out all necessary
precautions and regulations laid down for preventing the
spread of the disease.
In Italy the following usual infectious diseases have
to be notified : small-pox, scarlet fever, diphtheria, cholera,
measles, influenza, typhoid fever, erysipelas, typhus,
puerperal fever, and whooping cough, and any other con-
tagious or infectious disease that may appear in epidemic
form. We are also glad to find that in Italy the sanitary
authorities regard tuberculosis as an infectious disease, and
treat it as such, at least, disinfection is enforced after it,
although the pationt is not isolated.
If the doctor neglects to notify infections disease he is
liable to a fine of 500 francs, and in very serious cases he is
further liable to imprisonment in addition to the fine. The
latter is imposed under the penal code for personal damages.
This severity, however, is confined to the unfortunate doctor,
and with delightful inconsistence is not extended to the
Syndic, to which the real responsibility belongs, and which
can enforce precautionary measures or not, as it chooses, but
suffers no penalty for any neglect. The regulations recom-
mend, that in the event of infectious disease appearing in a
private house, the patient should be isolated as far as
Possible, and that afterwards room and clothes shall be dis-
infected. The sanitary offioial recommends what should, in
his opinion, be done ; but ho cannot compel his recommenda-
tions to be taken, unless ho can get the Syndio to back him
UP and give orders to that effect.
In nearly all Italian cities thero is a special building
adapted to receive patients suffering from infectious disease,
called a " lazz&retto," erected outside the town, or at least in
a thinly populated part. These are chiefly used in case of
epidemics. These buildings dato from the time when Italy
Used to be devastated by cholera.
Most of the hospitals in the larger cities are provided with
an entire wing which is used solely for infectious diseases,
^hia is notably the case at the principal hospital in Genoa
called the "Duchess of Galleria," where there is a large
delating ward for all cases of infectious disease, from outside,
v hen the patient is willing to go in and be nursed there.
These moat nearly resemble our special hospitals for infec-
tious disease in thia country. All smaller hospitals are pro-
vided with isolating rooms, to which any patients develop-
lng infectious disease whilst in the hospital are removed.
The weak point is that no authority, neither Syndic nor
medical officer of health, has any power to force a patient,
from whatever infectious disease he may bo suffering, to go
lQto an isolating ward or a lazzaretto to be nursed, if he does
Got wish it.
In the case of school children belonging to a family any
member of which is suffering from infectious disease, there
are no regulations to prevent such children going to school,
and so running the risk of carrying infection to their com-
panions. The teacher has no authoritative right to refuse
to admit such children, and can only do so on his own re-
responsibility.
As regards disinfection, the medical officer of health is
entirely responsible, and it is done under his direction, he
deciding the methods that he deems necessary under the cir-
cumstances. Thus we find that Bulphur is generally used for
fumigating rooms, and all linen is soaked in perchloride of
mercury. In the case of those who cannot affjrd to pay, dis-
infection is provided by the consumer, but there is no com-
pensation for olothing, &c., which is injured or has to be
destroyed. The loss, however, is usually made up by some
local charity.
Thus in Italy, though the regulations laid down in cases of
infectious diseases are good, the weak point lies in the carrying
out cf the precautionary and preventive measures. The chief
power in towns and districts rests with the Syndic instead
of with the medical officer of health, the latter thoroughly
realising the risks run, whilst the former has no special know-
ledge of sanitation, and the medical officer of health possesses
no power to enforce precautionary measures. His duty is
simply to notify to the Syndic, which, as a rule, pays no
attention whatever, nor do the higher authorities impose
any penalties on the Syndic for neglect of duty, though, as
already [seen, the unfortunate doctor suffers heavily for mere
neglect to notify. Moreover, what possible use is there in
mere notification, except to provide correct statistics of infec-
tious diseases for those who delight in figures ? The only
practical use of notification is that precautionary measures
may follow.
Another drawback is that the parish doctor is always also
the medical officer of health in small towns, and he is
always paid by the Commune, and thus his authority is
limited. If in any case he should appeal to a higher
authority for power to enforce sanitary measures whioh the
Syndic did not choose to carry out, the only result would be
that the Commune would dismiss him from his post. This
also applies to the medical officer of health in the larger
towns when he does not also combine the duties of parish
doctor.
The following is an example of what actually ocourred a
few years ago. Some isolated cases of amall-pox broke out in
the poorest part of the small town of N . The doctor at
once notified to the Syndic, and recommended that the patients
should be isolated. However, nothing was done. A few
more cases broke out, and the doctor again went to the
Syndic, and begged that a building might be fitted up tem-
porarily bb a hospital. The Syndic declared that they could
do nothing in the matter. A few more cases broke out, and
the inhabitants of the districb became alarmed. They
paraded before the Prefecture, armed with sticks and stones,
and threatened the Syndic. In their alarm the Syndic
sent for the doctor, and asked him what was to
be done, and he again propounded his scheme. Finally
the Syndic agreed to carry this out. The building was
fitted up with a dozen or more beds, and the doctor
represented to the patients ihat he wished to remove
them to a more healthy position where they could be more
easily nursed, and that for the sake of their neighbours and
relations they ought to go. All consented at once, and on a
fine warm night all were carried up to the isolating building
under the superintendence of the doctor. Night was chosen
so as to prevent a panic amongst the inhabitants.
Another instance occurred in Sicily, when a single case of
small-pox appeared; but the doctor could not induce the
Syndio to take any measures whatever, and it spread from
one to 120 cases.
Ixiv THE HOSPITAL NURSING SUPPLEMENT. May 13 1893.
pensions for IFlnrses.
The Statist, a journal for economists and men of business,
and one of the oldest and ablest of the weekly reviews,
published the following article in its issue of the 5th inst. :?
The Royal National Pension Fund for Nurses is regarded
with peculiar interest by business men. It is a child of the
City of London. It was founded in 1887 and endowed by
the generosity of a few City men with a sum of ?50,000.
Irreproachable as it is in its aims and conduct, its manage,
ment has been persistently attacked and its founder assailed
with gross personal abuse by two insignificant publications
. which pose as self-appointed champions of the nurses against
their best friends. Whether the editors of these papers are
women or men under petticoat influence, the style of the
attacks is characterized by feminine peevishness and
womanish want of logic and of knowledge oi accounts.
There is, moreover, an underlying spitefulness which can
only be accounted for by the existence of personal spleen.
The chief object of their abuse is Mr. Henry C. Burdett,
presumably because he was the happy originator of the
project, and carried it through without consulting the self-
important critic. His services on behalf of hospitals and
nurses warrant his treating such assailants with silent con-
tempt ; but the tactics lately adopted of spreading these
otherwise obscure periodicals gratuitously among City men
Bhow such a perverse animus that it may be worth while to
place before our readers a statement of the case in defence of
this valuable institution.
There are times in the life of many of us when we are in-
debted to the self-sacrificing serviced of the noble army of
nurses, and considering what a miserable pittance they
usually receive out of the remuneration which those, whose
gratitude they earn gladly pay, it would be a real satisfac-
tion to all to be assured that these handmaidens of humanity
are protected from want when worn out or past the age of
service. To ensure this is the one object of the National
Pension Fund, and, since none but the nurses themselves
can possibly make any gain out of the institution, it does
seem hard that it should meet with unreasoning opposition
and misrepresentation.
The querulouB criticisms may be reduced to three :?
(1) Thao the premiums charged are higher than those of
trading insurance companies.
(2) That the expenses of management are excessive.
(3) That the undertaking has been initiated and carried
out by benevolent and able business men, without the aid of
certain hysterical persons who assume to themselves a
monopoly in the affairs of the nurses.
The last, we suspect, is the true cause of all the outcry. The
work has been done quietly and well without the fussy
interference of the critics. Hinc illce lacrimal! But with
celestial passions and the resulting abuse we do not concern
ourselves.
Fictitious as the other two chargeB are, it may be well to
give a brief outline of the facts. It is true that the Council,
acting on the advice of their actuary, have, to ensure
absolute safety, fixed the premium at a rate slightly in
excess of those of several insurance companies, but inasmuch
as this rate is considerably below that charged for Po&t
Office annuities, it cannot be called unreasonable. More-
over, there are certain distinct advantages secured to nurses
by the fund which they would not get elsewhere.
(1) A member may at any time discontinue payments and
receive the whole of the back premiums with interest, the
fund thus serving as a savings bank.
(2) By making a email annual payment to the special Sick
Fund a member is entitled to an allowance wheaever she ia
laid up by illness.
(3j Members in distress have a claim on the Benevolent
Fund amounting to ?11,000, the gifts of generous donors.
(4) The whole of the profits, with considerable additions
from the Donation Fund, are shared among the members in
the form of a bonus added to the amount of the annuity.
On this point it is sufficient to quote a sentence or two
from the Bpeech of Mr. Walter H. Burns, the Chairman of
the Council:?
" These criticisms leave out of consideration the fact that
the Bonua Fund and all profits made are returned to the
nurses, in addition to what they pay for themselves; and
besides that, moat of the policies (those effected since 1890)
are on a returnable basis?that is to say, the nurses, or their
families, at death or before, can withdraw all the funds
which the nurses paid in, with interest at 2J per cent. All
the members of the Committee give their work for nothing,
and the disbursements are the mere actual outlays without
which the business could practically not be conducted. The
investigations to which I have been challenged by the critics
have only resulted in increased confidence in the soundness
and economy with which the fund is managed in the interests
of the class which it is established to benefit. If any company
charges apparently lower rates, it can only be an illusion, or
elsa it is done at a loss which cannot go on, for we are
organized on the basis of making no profit and tendering the
assured a substantial present besides."
The cost of management is always a weak point in benevo-
lent undertakings, especially in their early stages. The
amount under this heading in the accounts of the Fund is
certainly a large one, and yet, as the figures of last year
given below show, it would be difficult to single out any item
as extravagant in conducting so wide a business.
Expenses op Management.
Postages
Policy stamps
Stationery ...
Office chargos
Advertising ...
Auditors' fee
Sundries
Depreciation account
Salaries
Rent ...
? R. d.
97 11 4
68 8 2
150 9 0
99 3 8
77 15 0
21 0 0
81 14 7
8 6 9
678 1 4
105 0 0
t'1,387 9 10
The Council, doubtless, have their eye on this question, and
will gradually effect the necessary economies ; but, even as
the figures stand, almost the whole expense of management
is met by the surplus of the yield of investments at 4? per
cent, over the 3 per cent, basis on which the pension rates
are calculated. The nurses, therefore, receive back all they
pay, together with interest and a substantial present from
the Donation Fund besides.
It ia not surprising, therefore, that nurses turn a deaf ear
to misrepresentations, and are enrolling themselves in yearly-
increasing numbers as members of the Pension Fund. There
are already, after only five years' working, two thousand
four hundred members. A perusal of the report and the pro-
ceedings at the general meeting cannot fail to interest, an i
will prove to any impartial person the disinterestedness of
the Council and the immense importance of the work of the
institution.
There is one paragraph of the chairman's speech which
deserves a wider publicity.
" It is, perhaps, not out of place here to call attention to a
very touching instance of disinterestedness on the part of one
of ournurBes, Sister Emma Durham, who received ?200 for
services rendered as hospital-trained nurse to the late Lord
Tennyson, and has contributed the whole of the money to the
'Junius S. Morgan Benevolent Fund' for the sole use and
benefit of poorer nurses, in commemoration of the illustrioua
man whom she had attended in his illness."
It is for such women that the fund works, and whilst the
Council receive such practical proofs of a noble woman's
confidence, they can afford to disregard the captious criti-
cisms of envious detractors.
flDinor appointments.
Dudley Union Workhouse.?Miss Sophie Chllde has
been appointed Assistant-Matron at Dudley Union Work-
house. Miss Childe has previously held the posts of
industrial trainer at Sherborne Union Workhouse and that
of assistant-matron at the Union Workhouse, Dover.
Wants ant> Workers.
Old linen and left oil' clothes will be thankfully received 1 or lier poor
patients by the Distriot Nnrae, 82, Victoria Street, Aahton-nndcr-Lyno.
May 13, 1893. THE HOSPITAL NURSING SUPPLEMENT. lxv
a (Serman Iboapital.
By An English Patient.
III.?DAILY LIFE IN THE WARD.
To most people the idea of entering a hospital aa a patient
ia an unwelcome one. Preconceived ideas of the life suggest
dull monotonous dayB, rendered trying by the groans and
sighs of the other invalids. But that proves by no means to
be really the case. There are only one or two children
seriously ill, and the nurse, a lively creature, contrives to
make a great deal of fun even out of their fractiousnesa.
Most of the others are well, and cannot comprehend why
they must remain in bed three weeks. Everyone is ready
for a joke, and there are plenty of lively spirits amongst the
convalescents.
One afternoon, as I was dozing in blissful Ignorance of any
impending disturbance, my bed suddenly began to heave
like a ship at sea. I thought it was bewitched, and,
when sufficiently awake, I cautiously peeped under it and
discovered the spasmodic upheavals to be caused by one of
the convalescents, a regular madcap of a girl, ljing under
the bed and briskly jogging it up and down with her
shoulders, to the intense amusement of the others. This
joke, when quite new, was played upon one of the nurses,
and she, in utter ignorance of the cause of the disturbance,
screamed out, " Der Teufel, der Teufel, lieght unter meinem
bett!"
My next neighbour is a lively little girl of ten, who, while
we were both in bed, chattered and told me stories all day
long. She is one of five sisters, all of whcm are here. The
youngest is only cno and a half years of age, and was sent
along with her Bisters, not because she had the fever, but
because she was burdensome at home, as the mother was ill.
Naturally one would expect her to contract fever, but no,
she developed measles Instead, and was removed to another
ward. The day after another child followed suit, and in all
five or six were removed.
On Sunday and Wednesday afternoons, between two and half-
past four, the patients may receive visits from their friends.
As regards the infectious wards, strangers are not allowed to
enter, they may only survey their friends through the win-
dows of the door. Neither are infectious patients allowed to
walk in the garden duriDg visiting hours, but at all other
times, weather permitting, convalescents are expected to go
out regularly, having once received the doctor's permission.
The promenade is, of course, limited, because they are infec-
tious.
Before a patient is discharged the doctor writes what ia
called a history of the patient, the course the disease has
taken, any complications that may have arisen, diseases
already suffered from, whether the parents are still living,
aQd, if dead, of what they died. This paper, along with the
?yaterious one hanging over the bed, is then preserved in
the archives of the establishment for a period of from 25 to
30 years.
The nurses are on the whole a charming looking set of
S^la, so bright and fresh, some quite pretty. Those I see
are mostly the under-nurses, the head nurses cannot so well
leave their wards. According to the size of the ward varies
the number of nurses in it. This one ia of medium size, and
two nurses are attached. The neighbouring one is much
tafger, containing more than 30 beds, and in that there are
three nurses. The two nurses here have both been domestic
servants. The under-nurse is still nothing more than the
servant in the ward, with no responsibility and no nursing
to do worth mentioning. Besides doing most of the heavy
Work, it is her duty to fetch the meals from the kitchen, not
only for her own ward, but she has to take her turn with
other under-nurses in carrying food to other wards a.
certain times of the day.
The head nurse has been in the hospital many years, and.
is really very nice, although by no means refined. She has.
not a great deal of nursing to do, as the elder patients are
expected to be self-helpful from the beginning. Certainly,
aa far as the cure of the disease goes, Bhe cannot do muche,
that seems to rest with the patient herself. There is a little
girl of five here, who had nearly got ever the ftver when she
came, but she has a dreadfully swollen knee, which gives*
her great pain. After being here a lew days she began to be
hungry, and the doctor more than once said she could have
whatever she liked. Well, at ten o'clock the nurse gives her
a small egg, nothing more. After we have finished dinner,,
and it is nearly three o'clock, the nurse gives her a little
mashed potato and gravy, after which she gets nothing
unless she asks for it. Of course, she has her two portions,
of milk and some cocoa.
Nurse advises the patients not to tell the doctor of any.
slight pain. " No," she says, " the doctors don't like to be
bothered with every little ache and pain." .There is one girl
who allowed herself to be so persuaded. The pain became
daily worse, and at the end of a fortnight the doctor saw for
himself that something was wrong. The reiult was that she-
had to remain five weeks in bed. One can venture to suppose
that if the doctor had been told in the first instance, she-
would not have become so bad.
In the mornings, when the ward is being cleaned, two,
nuraeB are hardly sufficient, and if the convalescents did notr
help, the work would not be finished by ten o'clock. Con-
valescents are expected to make their own beds, and'
generally help in the ward, and being all young fervants th? y
are well able to do so. Moreover, every patient must re-
main at least six weeks in the hospital, three in bed, and'
three in the day-room, but they are quite well the last two-
weeks, and glad to help.
Each nurse has one dress a year of a dark grey material;,
also a dozen aprons and collars. They are not, however,
obliged to wear the dress every day ; at the same time, they
may not put it on when going out for their own pleasure.
Unlike English nurses, they have no distinctive outdoor
dress, neither do they wear caps indoors.
To every two or three wards there is an over-nurse, whose
grey dress and apron differ slightly from those of the other
nurses. Her duty, as far as I have observed, is generally to
superintend the wards in her charge, write upon the little
boards provided for the purpose the diet and medicine re-
ceived by each patient, to prepare everything for any
operation that is to take place in the ward instead of in tho
operating hall, and to weigh the patients. She also follows
the doctor when he makes his rounds in her wards, although
she is unable to answer many questions regarding the.
patients.
Twice a week nurses may go out from two o'clock p.m. till-
twelve at night, wli9n they are expected to be in again. Tho
head nurse must first ask the doctor's permission, which ia
given at once if there is no serious case in the ward; if there
is, she must then tell the over-nurse, who conveys the
information to the office.
When tbe under-nuree fetches milk or something extra in
the way of food which has been ordered for a patient, she
must first go to the office to get a paper, on which is written
exactly what she is to receive?how many portions of new and
how many portions of blue milk ; whether the fish is to be
boiled or fried. Unless this paper be produced the ccoks
cannot give the nurse anything. And if the paper does not-
bear the signature of one of the inspectors it is worthless.
In fact, I think they know to a crumb what is sent to the.
wards.
The nurses in the male wards are men, whose uniform ia &,
Ixvi THE HOSPITAL NURSING SUPPLEMENT. may 13,1893.
short white coat with bright buttons. The male over-nnrses
wear three-quarter length white coats, not quite so long as
the doctors. The same rules apply, doubtless, to these nurses
as to the females. I do not know accurately what the nurses
earn, but think pretty much the same as in England.
Amongst themselves the nurses always speak low German.
It is very ugly-sounding, but not difficult to understand,
many of the words being almost identical with English.
There is another important personage?there are three or
four of them in fact?of whom the nurses stand in awe, the
inspector. He is, indeed, one of the " powers that be," and
is liable at any moment to drop in and peer about; and woe
betide the nurse in whose ward a brass handle is unpolished
or the convalescents are indulging in too loud laughter.
Marntngs.
Under the heading, " An Insult to the Profession," The
Lancet inserts the following letter received by a doctor. We
cannot wonder at his indignation at the occurrence, and we
heartily wish that equal publicity were given to all similar
productions. There is another matter which ought to be
freely oondemncd and exposed by trained nurses when-
ever it takes place. We allude to the case of a medical man
offering a private nurse ?5 for every short and ?10 for each
long case put into his hands through her instrumentality.
Few private nurses feel sufficiently independent of their
employers to venture on exposing such things, or else they
shrink from incurring undesirable notoriety; but duty
should not be shirked, and unprofessional proceedings should
certainly be published as warnings, quite as much as the
accompanying letter, of which we and our readers would
gladly know the writer.
May 1st, 1893,
Sir,?I am at present engaged in organizing an institute
for the supply of nurses for medical, monthly, surgical, and
all other classes of cases. In order to gain the patronage of
medical gentlemen, I venture to offer them 20 per cent, of all
gross cash proceeds that come to me through their recom-
mendation. Allow me, sir, to express the hope that you will
do your best to enable me to secure the success of my venture.
I shall be happy to wait upon you at your convenience, to
give you any information you may require as to my arrange-
ments.
appointments.
Belfast Hospital for Sick Childeen.?Miss Aileen
Moriarty has been appointed Lady Superintendent of the
Belfast Hospital for Sick Children, Queen Street. Miss
Moriarty was trained at the Adelaide Hospital, Dablin, and
Pendlebury Children's Hospital, Manchester, and was after-
wards Matron of the Rous Memorial Hospital, Newmarket.
Such varied experience forms an excellent preparation for
the new work which Miss Moriarty is taking up, and we
wish her every success in it.
IRotes an& Queries.
Queries.
(125) Consumption.?Whero can I get special training for this branch
of nursing ? ?S. F. If.
(126) Mental.?Is there any bock published for asylum nurses??
Attendant.
(127) Dispensing. ? Where can a lady learn this in London??
Enquirer.
Answers.
' (125) Consumption (S. F. II.)?You will find a list of the Hospitals
for Diseases of the Ohest and for Consumption in " Burdett'a Hospital
Annual," 428, Strand, and then you had better write to the Matrons for
the rules for the training of probationers.
(126) Mental (Attendant).?There ia a useful book called " Mental
Nnrsing," by Dr. Harding, published by the Scientific Press, 428,
Strand,
(127) Dispensing (Enquirer).?See chapter on Dijpensing in Burdett's
" Hospital Annual." 428, Strand.
"Ibints for IRurses.
POULTICES FOR PRIVATE PATIENTS.
" In hospitals poultices are made in certain ways for the sake
of cleanliness and economy, and these ways are not always
the best possible for private patients, although they may be
the best under the conditions which obtain in hospitals," says
Dr. Lauder Brunton in " Disorders of the Digestion."
After explaining the effect of these applications, and the
difference between those used in medical or surgical cases,
he describes a special plan of his own for poulticing the chest
or abdomen, which is such an excellent one, that we quote
it at length, for the benefit of those nurses who have not read
Dr. Lauder Brunton's interesting work. He writes : "If
we apply the poultice directly to the skin, it must be allowed
to become tolerably cool before the patient can bear it, and
thus half its advantage is lost. In order to relieve spasm,
as in colic?intestinal, biliary, or renal?to relieve inflam-
mation of the pleura, the lungs, the liver, or other organs,
we want to apply the poultice as hot as possible, while we
protect the skin from being scalded. In order to do this a
flannel bag should be prepared, a convenient size being twelve
inches by eight, this should be closed at three edges and open
at the fourth ; one side of it Bhould be about an inch and a half
larger than the other, and it is convenient also to have four
tapes attached at the points which form the corners when
the bag is closed in order to keep the poultice in position.
Besides this, another strip of flannel should be prepared of
the same breadth as the length of the bag, and long enough
to wrap round it once or oftener. Crushed linteed, bowl, and
spoon should then be got together, and the spoon and bowl
thoroughly heated by means of boiling water ; the poultice
should then be made with perfectly boiling water, and rather
soft. As soon as it is ready it should be poured into the bag,
previously warmed by holding it before the fire ; the flap
which is formed by the longest side of the bag should now be
turned down and fastened in its place by a few long stitches
with a needle and thread, it should then be quickly wrapped
in the strip of flannel (also previously warmed) and fastened
in situ, if necessary, by means of the tapes. It may be
covered outside wilih a sheet of cotton wool. Ic this way the
poultice may be applied boiling hot to the skin without
burning; the two layers of flannel which are at [first dry
allow the heat to pass very gradually indeed to the skin; as
the moisture of the poultice soaks through them they become
better conductors, and the heat passes more quickly, but the
increase is so gradual as not to cause any painful sensations
whatever, but only one of soothing and comfort. The poultice
also naturally keeps much longer hot, and the necessity for
changing it arises much less frequently."
Mbere to (So.
Royal Albert Hall.?Free organ recitals every Sunday
afternoon, at three o'clock.
On Friday, May 26th, at quarter to eight, a lecture on
"Abernethy " will be given by Dr. Tom Robinson at the
Trained Nurses' Club, 12, Buckingham Street, Strand.
Tickets to non-members 6d. each.
The annual sale of work of ladies in reduced circumstances
will be held at the Portman Rooms, Baker Street, on Tues-
day and Wednesday, May 16th and 17th. The Duchess of
Teck will open the sale on Tuesday at three o'clock.
Lectures on Gynaecological Nursing.?Practical lectures
on the " Nursing of Gynaecological Cases " are given byjthe
physicians at the Women's Hospital, Chelsea, and nurses
from other institutions are invited to attend. Doubtless
many will be glad to avail themselves of suoh a privilege.
ZTbe Cbelsea Ibospital for Women.
An excellent concert in aid of this charity took place on
Tuesday last in St. James's Hall by the Stock Exchange
Orchestral Society. The choir of male voices and the
orchestra are especially to be complimented on the quality
of their performance. Sir Charles and Lady Halle and
several other well-known artistes lent valuable assistance.
We hope the financial results will prove as good as the
music throughout undoubtedly was.
May 13,1893. THE HOSPITAL NURSING SUPPLEMENT. ixvii
?ueen IDictoria Jubilee Jnstitute.
LECTURE ON NURSING IN CHOLERA BY SIR
JOSEPH FAYRER, K.C.S.I., M.D.
-St. Katharine's Royal Hospital, Regent's Park, is, as
most of our readers know, the headquarters of the Queen
Victoria Jubilee Institute, and a very quaint and picturesque
centre it is.
On Thursday afternoon, May 4th, the long narrow hall
was closely packed by nurses in the distinctive cloaks and
bonnets with which we have all become familiar, and the
pretty badge which is everywhere recognised as betokening
a regularly appointed " Queen's Nurse." There were several
superintendents present, and as the clock struck the hour
fixed for the lecture, H.R.H. Princess Christian arrived, and
took her place near Sir Dyce Duckworth, who officiated
as Chairman, in the unavoidable absence of the President,
Mr. Peile.
Sir Dyce Duckworth, in introducing Sir Joseph Fayrer,
spoke of the great advantage which it would be to all present
to listen to a lecture from so experienced a doctor, and one
moreover who had spent so many years in the very home of
the cholera germ, viz,, the region of the Ganges. He deeply
regretted the absence of the President, and referred to the
interest in this important subject displayed by Her Royal
Highness, who was now honouring them by her presence.
Sir Joseph Fayrer quoted at some length from an ad-
mirable lecture previously given to the nurses by their
President, and he also commended the rules (given below)
formed for their guidance in case of an epidemic of cholera.
He spoke of cholera as an emergency which menaced
England, and had already visited other lands. The lecturer
insisted on the fact that to be forewarned should also mean
to be forearmed, just as peace was best ensured by a nation
which was prepared for war. He dwelt specially on the
necessity for all preparations being done quietly and unosten-
tatiously, as panic was, above all things, to be avoided.
The general improvement of local sanitation was one
of the best preparations which could be made, and
Sir Joseph Fayrer thought the steps that had been
taken by the Local Board were so far excellent. How-
ever, the fact still remained that an enormous number of the
dwellings of the poor were still overcrowded and unsanitary,
and these two conditions were the most disastrous ones pos-
sible. District nurses could do much by example as well as
precept in improving the condition of the poor, and in
inculcating the necessity for pure drinking water, free
ventilation, &c.
More than once during the afternoon the lecturer urged
upon his hearers the absolute need for immediately summon-
ing medical aid to every case of cholera, however apparently
slight its onset might be. No nurse should depend on herself
alone in this matter. Sir Joseph Fayrer spoke in very high
terms of the extraordinary progress in nursing which had
taken place in England during his absence in the East. No
social movement had been more beneficent than the one which
had culminated in the present remarkably perfect syBtem of
nurse training.
With regard tj cholera, early measures were of the greatest
value, and these could often check or even ward off an attack,
therefore he made a great point of the "first aid," which,
in the hands of competent women, would be ancillary to the
medical treatment.
Above all things, nurses Bhould be ready, willing, and able
to recognise cases at the incipient Btage, and this would be
no encroachment on the physician's duties.
As regarded the infectious character of the disease, Sir
Joseph impressed upon his audience his own emphatic opinion
that the attendants on cholera cases ran no special risks, and
were certainly not more likely to be attacked, than any mem-
bers of the general public.
In quarantine he had not the slightest confidence. Good
sanitary local precautions were of much greater value, and
alao the maintenance of good general health and even spirits.
Chills, over fatigue, and depression were predisposing
causes, and so were over-indulgence in irritating foods,
BtimulantB, over-ripe fruit, &c.
It was part of a nurse's duty to guard against lowness of
spirits, and also against excessive excitement, in fact, an
equable state of mind was the one with which cbolera could
be faced most securely. Panic was the friend of disease, and
therefore must be avoided, and great pains should be taken
not to allow people to become either discouraged or
depressed when an epidemic of this mysterious pestilence
threatened.
Sir Joseph Fayrer gave his hearers to understand that he
had good hope that England might escape again this year
from an epidemic of cholera.
The usual directions with regard to disinfection, fumiga-
tion, &c., were given, and also advice as to the boiling
of water, and of milk shortly before use.
The necessity for promptly checking diarrhoea in cholera
seasons was urgently insisted upon, and Sir Joseph said that
although such a step might occasionally be harmful, the
danger would be inappreciable as compared with the risk of
allowing it to continue and to develop into an attack of
cholera.
Various astringent remedies were given, but the nurses
were counselled to learn and strictly adhere to the
rules laid down by the doctors in whose districts they
worked. The lecturer said he had a great distaste to
addressing the general public, but he could not class his
present audience in that category, and he had every
confidence in the intelligent co-operation of those to whom
he spoke.
Recommendations of the Council to Superintendents
of Nursing Homes Affiliated to the Institute, in
View of the Threatened Outbreak of Cholera.
I.
The Council recommend that Superintendents of all
Nursing Homes affiliated to the Institute should without loss
of time acquaint themselves "with what (if any) special
arrangements have been made for giving medical assistance
within the district,"* with a view of learning from the sani-
tary authority of the district what steps shall be taken by
nurses with regard to any cases of diarrhoea or cholera which
(in the event of an epidemic) shall come under their notice ;
more especially as concerns the notifying and removal of
such caseB to a hospital.
II.
That having obtained this information Superintendents
should inform every nurse :?
(?) How to communicate with least possible delay with
the proper medical authority for notification of any
case which may come under her notice.
(?) As to the nearest centre for obtaining medical help,
medicines, disinfectants, &c.
(c) As to the regulations of the Local Government Board
or sanitary authority of the district with regard to the
disinfection of houses, furniture, &c., so that Bhe may,
if needful, at once take the necessary steps to obtain
such disinfection.
(d) As to the arrangements for affording relief in the
districts that the nurse may, without loss of time,
communicate with the relieving officer, or other person
appointed to dispense such relief in any case of want
or destitution which may come to her knowledge, Bince
"privation, as predisposing to disease, may require
special measures of relief." *
III.
That in the event of an outbreak of cholera, Superin-
tendents of all Nursing Homes affiliated to the Institute should
as far as possible observe the following precautions with
regard to their nurses :?
(a) That no nurBe go on duty if over-tired or unwell,
especially if Buffering from loosenesa of bowela or
diarrhoea.
(b) That an extra allowance of nourishing food be pro-
vided for any nurae obliged to work beyond the usual
hours, and especially that such food be available for
any nurse called upon to work of a night.
(c) Thateyery nurse be specially cautioned against any
neglect in the matter of scrupulous cleansing and dis-
infection of her hands and clothing after dealing with
a case of cholera.
* General memorandnm on proceedings which are advisable in places
attacked or threatened with epidemio diseiae.?Local Government
Board, August 26th, 1892.
lxviii THE HOSPITAL NURSING SUPPLEMENT. May 13, 1893.
XTbe flDuses' %oofttno (Blass.
PICTURES.?THE NEW GALLERY.
By C.
Of all the galleries in our city, the New Gallery stands first
on its own merits, and this year I think it can stand doubly
secure by reason of the merit of its pictures also. Many of
us might hesitate twice before entering the Academy on a
hot day. The New gallery, on tbe other hand, lures
us with a promise of coolness, quiet, and a not too
bewildering number of pictures, amongst which we are
sure to find some favourites which can be dwelt upon
without tco great a crowd intervening between us and the
picture. Sit down then a moment in the cool entrance hall,
with the pleasant splash of the little fountain, and coon the
spirit will move you to explore the treasures on the walls of
the rooms and balcony.
Entering the large room which is opposite, and proceeding as
the numbers run, the first picture that attracts our attention
is a portrait of Mr. Gladstone by Sydney Hall (22). Mr.
Gladstone is represented standing at the lectern in Hawarden
Church reading the lessons. The portrait is small, but a
striking likeness of the Premier,with a serenity and earnest-
ness of expression which gives a pleasing impression to the
beholder, and is in keeping with the scene of which he forms
the centre.
Arthur Hacker's portrait of a little boy which occupies a
central place on wall No. II. of this room is a delight-
fully natural and graceful representation of a child.
The child's face is fresh and bloom - like in tone, and
well thrown up from a happily chosen back-ground.
On the same wall furiher on is a portrait which I think
you will agree with me is a charming portrait of an old
lady. The dainty Quaker-like cap and costume, and the
dear old hands folded in front of her are quite lovingly
depicted by the artist. Before I finally left the gallery I
went back and looked at my old lady again, with her cheeks
yet fresh with colour, and her kindly blue eyes.
No. 71, "Sweet April" by James Grace, cannot fail to
delight lovers of landscape. The many beauties of the
picture unfold gradually aa one gazes. Sweet April is throw-
ing off the chilly grey and red brown cloak of winter and the
fairy green of the birches is asserting itself in the copse to
the left of the picture. In front is a mirror-like
pool, out of which tall rushes grow. High banks of rich brown
earth are the confines of the scene ; these are crowned with
the budding gold of the gorse ; the distance melts in soft
grey blue. I think of all the landscapes in the gallery this
gave me the greatest delight to dwell upon. I cannot convey
with feeble pen one little shadow of what the painter has
expressed with skilful brush. Sit for yourselves before the
picture, it can so ably speak for itself. Beyond my favourite,
which I left with reluctant steps, is a panel of wonderful
blue. It is entitled, " Neptune's Horses." These sea-born
steeds reveal themselves as we gaze, out of the foam and the
blue of the wave, this effect of the artist is a pretty conceit,
a good interpretation of so fanciful a theme.
When I had proceeded so far on my journey, the remarks
of two clergymen, who spoke in very loud tones, began to
attract my attention. They stopped in front of a portrait
of Canon Mason, and drew attention to the small crucifix in
the background of the picture. They said much on the
scandal of a clergyman of the Church of England appearing
in the vicinity of what they teemed to regard as a Jesuitical
emblem. Later on they stood before the portrait of another
dignitary of the Church. Here also they did not seem satis-
fied either. " Terribly broad views, terribly broad," one re-
marked ; " high time he was removed from ." This is
the spirit in which some of us patronise art exhibitions ! But
to return to our pictures in the first room. On the last wall is a
very interesting picture of two boys, by Herkomer (No. 92).
They are two nice-looking, manly fellows in Highland
costume. One sits amongst the bracken and verdure of the
heathland with a rabbit in his arms. The brother stands
beside him. The background is a fine belt of trees.
" The Tramp," further on the sime side (No. 96), is a
sadly true picture of a poor, hunted-looking woman and her
child, resting by the hill side. Near the door is a weird
picture of " The Man with the Iron Mask " in his cell, by
Philip Burne Jones. The whole tone of the picture is dim
and green, and suggestive of the despair and wretched loneli-
ness of the armoured figure surrounded by prison walls. It
is a relief to turn to a clever little picture at its side (No.
105), called " I Don't Believe it," by Miss Miriam Davis.
Nothing but the bare interior of a room and two women
gossiping, but a clever little picture withal, and one pos-
sibly somewhat overlooked by reason of its size and high
position.
Now we come to the second room. The first wall here
boasts of four large portraits. The most striking of these
is of Mrs. Hammersley, occupying the centre of the wall.
She is dressed in a gown of the most brilliant magenta-
pink velvet, seated on a couch, a back-ground of white-
grey cut tain. Tnis bright gown, against the surrounding
of grey, is most effective. The whole effect of the
portrait is animated and striking. My clerical critics
were not of my mind, however ; they surveyed the picture,
pronounced it terrible, and declared Mrs. Hamtnersley's feet
were Japanese.
I am bound to admit that such colouring as Mr. Sargent
has displayed in his picture proves a little hard on unfortu-
nate neighbour pictures. The landscapes which hang side
by side to it acquire an unnecessarily sad aspect, in conse-
quence of its vicinity. "Cain's First Crime," hangiDg
on the same wall, is represented by Mr. Kennedy in a.
rather inexplicable manner. My clergy could not under-
stand it at all, bo how can I venture to explain ? Adam and
Eve are represented in the Garden of Eden, grouped beside
a stream. Cain is presenting an apparently live lizard to a
Btork-like bird in front of him. Was this the crime ?
The next wall is largely occupied by a tiger scene. "Dip-
ping for Sprats " further on (186) is a pretty picture of a boat
with three figures in it. Two boys are dipping their nets into
the green sea, whilst the man steadies the boat with the oars.
In the distance is a flotilla of fishing smacks.
The last wail possesses a fascinating delineation of the
dearest little flaxen-haired boy on the brow of a hill, whilst
huntsmen wind round the hill below. Over the door is a.
lifelike bit of woodland scenery.
Entering the third room three central pictures strike one
at once. The first of these, on the left side of the room, is
another picture of " Neptune's Horses," by Walter Crane.
In this picture Neptune himself is visible, driving his horses-
in line before him. They bound over the crest of the wave,
and dash onward towards the shore. To me the vague treat-
ment of the same subject in room two appears far more poetic
and suggestive. The second central picture, hanging on the
next wall, is No. 233, "Gold, Incense, and Myrrh." The
figures present a weird, ethereal effeot in the moonlight, and
the whole picture is suggestive of some mysterious heathen
rite rather than the adoration of the Magi. On each side of
this picture are some delightful companions. One (231) shows
us a barge passing under a bridge over the canal in the
midst of a town. On the bridge is a stream of passers-by,
and on the barge is the bargee's family engaged in navigating
the barge through the bridge. The picture is full of life and
colour. No. 234, " At Set of Sun," by David Murray,
shows some beautiful kings of the forest?pine trees?witb
their trunks ruddy in the light of the setting sun, and their
dark forms strongly defined against a background of light.
The third central picture on the following wall is No. 274,
" A Greek Lemon Gatherer." Such a very green and yellow
picture this, that our English eyes are inclined to question an
effect unfamiliar to many of us. But the artist, after
all, has not produced a scene more vivid than can be
beheld by any wanderer beneath a lemon grove with the
sun shining strong above. A lemon grove is far more
beautiful and graceful than the more talked of orange
grove, and Mr. Tuke evidently very much fascinated by
the subject, has another similar study of lemon tree?
in the same room.
There now remains but the balcony, which runs round the
Central Hall, to^be seen. Here are placed the water-colour?
and smaller pictures, many of which are charming
little gemB. Space compels me to pass over them briefly.
Numbers 283, 297, 306, 318, and 348 struck me especially.
" Salisbury," which has charmed so manv artists, is
delightfully represented by Mr. Whipple (282). " Tho
Children of the Marquis of Cholmondeley " form a delightful
little picture by Miss Holland. The balcony is not the
least interesting part of the collection. I cannot too warmly
advise those who have not visited the New gallery to do so.
I think they will feel with me that it is a most refreshing
treat that will afford many a pleasant memory
May 13 1893. THE HOSPITAL NURSING SUPPLEMENT. lxix
Zbe 36ook TKHorlb for Women anfc Burses
?We inyite Correspondence, Criticism, Enquiries, and Note' on Books likely to interest 'Women and Nurses. Address, Editor, The Hospital
(Nurses' Book World), 428, Strand, W.0.1
Tennyson, Ruskin, and Browning. By Anne Thackeray
Ritchie (Macmillan and Co.)
The proportion of "Reminiscences" which make good
reading is very small. Deducting a few good stories which
may be found embedded in most of these publications, now
become bo tediously fashionable, they arc found for the most
part to be composed of the dreariest padding. And when
the dull talk fastens on names we hold in reverence, it is
liable to become "most tolerable, and hardly to ba
borne." But Mrs. Ritchie sheds over the scenes which she
revives a delicate aroma entirely her own. Her pages present
a delicate series of pictures which charm by a hundred
indefinable touches. And these pages will call up for many
readers, ill-disposed towards reminiscences in g-sneral,
pleasant images not soon to fade away, which may help
them to piece out half-formed impressions. Mrs.
Ritchie's gift for setting an atmosphere before her
readers in a few words is unique. " I can remember
on one occasion, through a cloud of smoke looking
across a darkening room at the noble grave head of the
Poet Laureate. He was sitting with my father in the
twilight, after some family meal, in the old house in
Kensington. It is Lord Tennyson himself who has reminded
me how upon this occasion, while my father was speaking to
cti?, my little sister looked up suddenly from the book in
which she had been absorbed, sayiDg in her Eoft, childish
voice, " Papa, why do you not write books like ' Nicholas
Nickleby?'" Here is the description of Tennyson's
reading. " Reading is it! One can hardly describe it. It
is a sort of mystical incantation, a chant in which every note
rises and falls and reverberates again. As we sit around the
twilight room at Farringford, with its great oriel window
looking to the garden, across fields of hyacinth and self-sowed
daffodils towards the sea, where the waves dash against the
rocks, we seem carried by a tide not unlike the ocean's sound ;
it fills the room, it ebbs and flows away ; and when we leave
it is with a strange music in our ears, feeling that we have
for the first time, perhaps, heard what we may have read a
hundred times before." The talk about Ruakin will interest
many readers, but the element of personal recollection in it is
small. Of Mr. and Mrs. Browning there are very happy impres-
siona, the haza of distance hanging about the scenes through
whichglimpses of the well-known figures shine out. Here is one
of these little word pictures. Mr. and Mrs. Browning are
discussing spiritualism with Thackeray. " Mrp. Browning
believed, and Mr. Browning was alsvays irritated beyond
patience by the subject, I can remember her voice, a sort of
faint minor chord, as she, lisping the 'r' a little, uttered her
remonstrating " Robert,"and his loud and dominant baritone
s weeping away every possible plea she and my father could
make; and then came my father's deliberate notep, which
seemed to fall a little sadly?his voice always sounded a
lxx THE HOSPITAL NURSING SUPPLEMENT. Mat 13,1893.
THE BOOK "WORLD FOR WOMEN AND NURSES-continued.
little sad?upon the rising waves of the discussion. I can see
Mr. and Mrs. Crowning with their faces turned towards the
window, and my father with his back to it, and all of us
assembled in a little high-up room. Mr. Browning was
dressed in a rough brown suit, and his hair was black hair
then, and she, as far as I can remember, was, as usual, in soft
falling flounces of black silk, and with her heavy curls drooping,
and a thin gold chain hanging round her neck.'' Can we
not all see it too and hear the voices and taste the essence
of it? We have purposely chosen these little scenes as
specimens of Mrs. Ritchie's peculiar art. But for those
who like something more definite, there is great plenty of
anecdote and biographical detail, with many hitherto
unpublished letters which greatly add to the value of a very
charming volume.
Old-fashioned Education.
Writing in Longman's Magazine for April, Mrs. Lang
gives a lively criticism of that old nursery bug-bear, the
Fairchild Family, with a short biography of the authoress,
Mrs. Sherwood. In all the deadly tedium inflicted by Mr.
and Mrs. Fairchild it has always been assumed that they
were admirable parents. Mrs. Lang, however, boldly
Bhatters this image, and her diaseotion of the Fairchild
system of education opens up a new view of the matter.
"To make up for the lack of worldly instruction it was Mr.
Fairchild's habit to give a singular sort of object-lesson to
his family whenever the opportunity permitted. He seized
the excuse afforded him by a childish quarrel between Lucy
and Emily to escort them all three to a wood a Bhorb distance
off, where the body of a man was still hanging from a
gibbet. He theninsistedonsittingdownclose underthe gibbet,
with its rattling burden, and giving them the whole history
of the bones that were swinging above their heads, and of the
envy and jealousy that had finally placed them there. Not?
long after this ghastly episode follows another, still
more revolting. An old man in the village dies, and Mr.
Fairchild remarks to his children, ' Have you any desire to
see the corpse, my dears ? You never eaw ajcorpse, I think ? '
To which Lucy answers, ' No, papa ; but we have a great?
curiosity to see one.' Accordingly, after summoning Emily
and Lucy to repeat all the texts they could remember about
death, they proceed to the cottage and are invited in.
' When they came to the door they perceived a kind of
disagreeable smell, such as they had never smelt before;
this was the smell of the corpse, whioh having been dead now
nearly two days, had begun to corrupt.' It is incredible
that any man should voluntarily have exposed children to
such an ordeal; still more that he should have kept them
there a considerable time. It is a wonder they were not
made physically ill or else frightened into fits; but the
family were unusually stolid ! " It is amazing after this to
read the account of Mrs. Sherwood's useful life and varied
experiences, and to learn that she had an excellent education
and was accustomed to mix in good and cultivated society.
The system of education in'common vogue in her day must
indeed have been remarkable when the Fairchild methods
were hailed as an ideal.

				

